# Contracting retail pharmacies as a source of essential medicines for public sector clients in low- and middle-income countries: a scoping review of key considerations, challenges, and opportunities

**DOI:** 10.1186/s40545-023-00557-w

**Published:** 2023-05-02

**Authors:** Warren A. Kaplan, Carlotta M. Cellini, Kwesi Eghan, Kevin Pilz, Denise Harrison, Veronika J. Wirtz

**Affiliations:** 1 Department of Global Health, Boston University School of Public Health, 715 Albany St, Boston, MA 02118 USA; 2 Management Sciences for Health, 4301 North Fairfax Drive, Suite 400, Arlington, VA 22203 USA; 3 USAID, 300 Pennsylvania Avenue, Washington, NWDC 20523 USA

**Keywords:** Public–private contracting, Retail pharmacies, Essential medicines, Health system strengthening, Health system administration, Low- and middle-income countries

## Abstract

**Background:**

Insurances in high-income countries (HIC) often contract with private community pharmacies to dispense medicines to outpatients. In contrast, dispensing of medicines in low- and middle-income countries (LMICs) often lacks such contractual arrangements. Furthermore, many LMICs lack sufficient investment in supply chains and financial and human resources to guarantee stock levels and services at public medicine-dispensing institutions. Countries striving to achieve universal health coverage (UHC) can, in principle, incorporate retail pharmacies into their supply chains to expand access to essential medicines (EMs). The objectives of this paper are (a) to identify and analyze key considerations, opportunities and challenges for public payers when contracting out the supply and dispensing of medicines to retail pharmacies and (b) to provide examples of strategies and policies to address these challenges.

**Methods:**

A targeted literature strategy was used to conduct this scoping review. We created an analytical framework of key dimensions: (1) governance (including medicine and pharmacy regulation); (2) contracting (3) reimbursement; (4) medicine affordability (5) equitable access; and (6) quality of care (including ‘patient-centered’ pharmaceutical care). Using this framework, we selected a mix of three HIC and four LMIC case studies and analyzed the opportunities and challenges encountered when contracting retail pharmacies.

**Results:**

From this analysis, we identified a set of opportunities and challenges that should be considered by public payers considering public–private contracting: (1) balancing business viability with medicine affordability; (2) incentivizing equitable access to medicines; (3) ensuring quality of care and delivery of services; (4) ensuring product quality; (5) task-sharing from primary care providers to pharmacies and (6) securing human resources and related capacity constraints to ensure sustainability of the contract.

**Conclusion:**

Public–private partnerships offer opportunities to improve access to EMs. Nonetheless, managing these agreements is complex and is influenced by a variety of factors. For effective contractual partnerships, a systems approach is needed in which business, industry and regulatory contexts are considered in tandem with the health system. Special attention should be devoted to rapidly changing health contexts and systems, such as changes in patient preferences and market developments brought about by the COVID-19 pandemic.

## Background

It is common for retail pharmacies in high-income countries (HIC) to have a contract or an agreement with public or private insurances to dispense essential medicines (EM) to outpatients [1]. This is in stark contrast to many low- and middle-income countries (LMICs), where such contracting or outsourcing often does not exist [2]. The WHO has recognized retail pharmacies as important partners for LMICs to expand the services traditionally provided by the public sector. This is partly because private sector pharmacies offer client service delivery advantages (e.g., more convenient locations and more consistent stock) which may translate into financial savings and improved retention in care [3, 4]. Furthermore, public sector-operated supply chains continue to face challenges in ensuring continuous availability of EM and other health products [5, 6]. For instance, the limited availability of FP as essential health supply in the public sector is one reason for the unmet need for FP; about 25% of women in their reproductive years in Sub-Saharan Africa are estimated to have unmet FP needs [7]. Incorporating retail pharmacies as an integral part of the public health system supply chain thus presents itself as a unique opportunity for countries striving to achieve universal health coverage (UHC) and implement insurance and other medicine benefits schemes.

The objective of this paper is to describe the key considerations, challenges, and opportunities for LMIC public payers when contracting retail pharmacies to provide essential health supplies to public sector clients.

## Methods

This study was part of a larger USAID Medicines, Technologies, and Pharmaceutical Services (MTaPS) program project on strengthening pharmaceutical management (contract no. 7200AA18C00074).

### Case study selection

We identified and reviewed documents related to public payers in HIC and LMICs contracting with retail pharmacies to serve public sector clients for the receipt of EMs, FP, and other essential health care products. Informed by this initial review, we selected seven country cases based on the following criteria: (1) evidence of a formal contract or agreement between retail pharmacies and the public payer; (2) an agreement on the provision of EMs or reimbursed medicines including FP; (3) a mix of high, middle and, wherever possible, low-income countries with single or multiple public payers. In addition, each case study’s contract or agreement with the private sector (4) must have been in place longer than 1 year and (5) must have the documentation of the contents of the contract or agreement in the public domain. We used the 2021 World Bank country income classification to define HICs and LMICs [8].

We selected Brazil and Argentina as upper-middle-income countries, Ghana and Namibia as lower-middle-income countries, and Spain, Sweden, and the United Kingdom (mainly England) as our high-income countries. We note that despite Sweden not meeting the first inclusion criteria of having a formal public–private contract, it was included because of its relevant history and experience in changing from a public-owned pharmacy model to one that allows private ownership [9].

### Literature review

We employed a targeted literature strategy to conduct a scoping review. The question we sought to answer was: What are the promising policies and strategies for outsourcing the delivery of EM for public sector clients to retail pharmacies? What are key considerations, challenges, and opportunities for public payers when contracting-out the dispensing of Ems including FP to retail pharmacies?

**Fig. 1:**
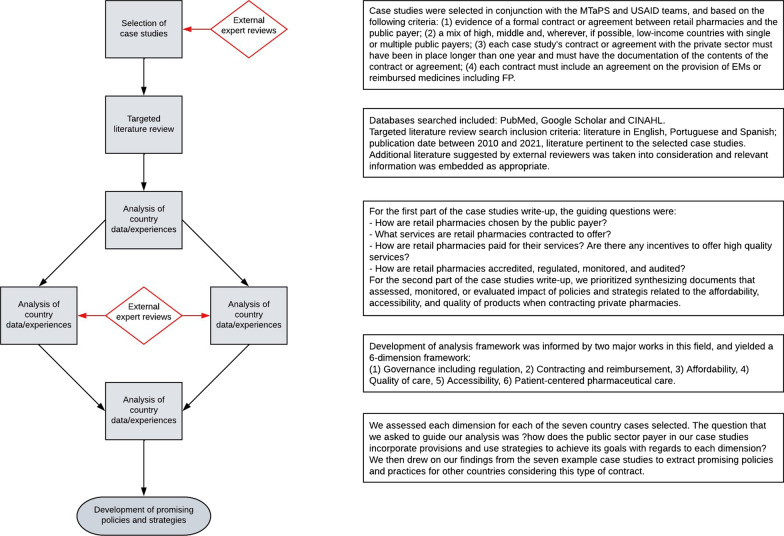
Methodology flowchart

The search criteria included literature in English, Portuguese and Spanish and we limited the search to the last 12 years (2010–2021). See Appendix 1 (Fig. 1) for a more detailed search strategy, data extraction and analysis, a description of keywords used, and a list of references for each case study.

We identified several policy domains in the literature [10] and disaggregated them into an analytical framework consisting of six key dimensions: (1) governance (including medicine and pharmacy regulation); (2) contracting (3) reimbursement; (4) medicine affordability; (5) equitable access; and (6) quality of care including those related to ‘patient-centered’ pharmaceutical care. This framework summarizes the key considerations for public payers when contracting the private sector to deliver FP products but is also very relevant to EMs more broadly. We extracted information according to the domains in our analytical framework.

We compared and contrasted the findings of each case study, and, based on our framework, we drew conclusions and recommendations for those public payers thinking of contracting with retail pharmacies. We considered contextual factors, such as the wholesaler and distributor market, business regulations, and incentive structures.

## Results

Table 1 summarizes the key dimensions that were considered in our analytical framework and the related key opportunities and challenges for the public sector contracting of retail pharmacies.

**Table 1:** Key dimensions, related challenges, and opportunities with regard to public-sector contracting of retail pharmacies

Key dimensions	Key challenges	Opportunities to address challenges
Governance and regulation of the contract	Balancing business viability with affordability of goods and services	Contract-based business incentives, e.g.: • Unit responsible for setting reimbursement prices; • Participation of pharmacies in public procurement; • Support from Pharmacy Services Administrative Organizations (PSAO) • Digital tools to support retail pharmacy management, etc.
Accessibility	Incentivizing geographically equitable access to medicines	•Procurement and health insurance policies that support local supply chains and pharmaceutical markets; • Provisions for stock financing to grow accessibility
Quality of Care	Ensuring quality of products, care, and delivery of services	• Supervisions, audit, and inspection of private sector quality of products and care
Patient-centered pharmaceutical care	Task-sharing from primary care providers to pharmacies	• Financial incentives to retain qualified personnel • Training and continuous professional education for pharmacy staff
	Human resource and capacity constraints	

With respect to opportunities, contract-based partnerships between public payers and the private sector offer a pathway for increasing access to medicines. For example, financial incentives have been provided to retail pharmacies to obtain medicines inexpensively and some countries have even managed to provide reimbursement prices at a level sufficient to incentivize the private sector to have a viable business model. For example, in Spain, all outpatient medicines are included in a regressive margin system [11].

These types of incentives can also promote access to medicines in rural communities. Parts of the UK provide funds for this purpose and providing criteria for new pharmacies based on distance between pharmacies or number of people served [12]. Spain creates a market for new retail pharmacies/licenses and has created specific criteria to open up new establishments in rural areas [13].

Product and service quality can be successfully supported via the appropriate use of information technology (IT) systems, as is the case in Argentina, or accreditation, as is the case in Brazil [14, 15].

Improving the affordability and availability of medicines also plays a critical role in increasing access to medicines. Since 1999, the dispensing of generic medicines has been prioritized in Brazil, meaning that providers, including private retail pharmacies, are required to first dispense these products if possible [16]. This strategy has encouraged the use of quality-assured medicines at lower costs and has contributed to a significant increase in the availability of certain medicines, particularly those for hypertension, diabetes, and asthma [17].

Ghana introduced a single National Health Insurance Scheme (NHIS) benefit package covering 95% of disease conditions and placed few limits on NHIS members (e.g., no cost sharing beyond premiums and no annual or lifetime limits). NHIS allows contracting with retail pharmacies to deliver some of the benefits and this has improved affordability, adherence, and quality of care [18].

Task-sharing and the need for pharmacies to have more primary care responsibilities are being recognized, and Sweden has created a pilot program to investigate this. In the UK, there is strong economic evidence to suggest that for people with or at risk of acute illness and medical emergencies, community pharmacy-based enhanced services are cost-effective. The most recent NHS contract in the UK considers the need for pharmacies to engage with local Primary Care Networks. These interventions reflect certain advanced services currently provided at community pharmacies in England, such as community pharmacist consultation, flu vaccination, hypertension case finding, etc. [19]. These pathways, discussed in more detail below, are not without challenges.

### Challenge 1: balancing business viability with affordability

Our review highlights the difficulty in balancing public and private sector financial viability with the affordability of products offered to patients by retail pharmacies. The Ghana and Brazil case studies illustrate that when public sector reimbursement to private sector retail pharmacies is lower than the pharmacy acquisition price—private pharmacies take a loss and are thus less attracted to enroll in the public–private contract [20-22]. In the Ghanaian case, this was due to retail pharmacies incurring additional recurrent and capital expenses, whereas public pharmacies’ expenses were paid by the government. Moreover, public facilities benefit from lower prices of medicines made possible by competitive tender purchasing provided by the central and regional stores, which private Ghanaian pharmacies were unable to participate in [20]. On one hand, if the reimbursement margin is low, private sector providers may incentivize users to increase the consumption of their products and services, which, in turn, can have a negative effect on health or result in a waste of resources. On the other hand, private pharmacy providers may decide to stop providing services under the public system altogether. In some instances, private pharmacies may even act outside the contracts by requesting patients to pay the difference between the current market prices and reimbursement prices.

In Brazil, under the Farmácia Popular Program (FPP), the Ministry of Health (MoH) sets reimbursement prices for a limited list of medicines to be offered at accredited private pharmacies contracted under the program. Specifically, the government agreed to pay up to 90% of the reimbursement price [23]. If the selling price is higher than the reimbursement price, the rest of the cost is the responsibility of the patient. Nonetheless, an audit report pointed a significantly higher price of 13 medicines in the retail pharmacies portion of the program compared to the public sector. For example, in 2018 insulin was 2.5 times more expensive in the AFP than in the public sector. Because of the financial risks this posed to patient affordability and access, 5% of private pharmacies were removed from the program [23]. The Brazilian government also decided to make a restricted number of chronic disease medicines (for hypertension, diabetes, and asthma) free of charge in retail pharmacies. [24].

Sweden introduced a substitution policy (i.e., pharmacies being allowed to exchange medicines for less expensive alternatives; this procedure is commonly called “Products of the Period”). Briefly, under this program, the government determines which generics are exchangeable. Such exchange groups are selected monthly. To be able to participate in this pricing and purchasing mechanism, the company with market authorization of a “selected product” is required to enter a contract in which it attests to specific stipulations regarding its financial and logistical capacity. The final selection mechanism uses a reverse auction approach in which the company with the lowest bid is selected [25, 26]. This exchange/auction system has its own advantages and disadvantages. The system provides lower prices and more affordable medicines. Each month, new forecasts must be made, and stocks filled with the newly selected generic medicines that will be exchanged. Managing all of this is considered time-consuming and inefficient because of the logistical support needed to cover several deliveries of potentially new products each month. There is also a perception that some pharmacy chains are prioritized more than others by the wholesalers, resulting in backorders at some specific pharmacies [25].

Providing financial incentives for retail pharmacies to obtain medicines inexpensively faces many challenges. Public payers typically use reimbursement prices as a measure of cost containment. Some countries have successfully set these reimbursement prices at such a level that it incentivizes businesses to obtain medicines at a low price, but still be able to remain financially viable. This system is not fool-proof; for example, in the United Kingdom (UK), pharmacies are incentivized to obtain generic medicines inexpensively as they keep any difference between their procurement price and the National Health Service (NHS) reimbursement price [27]. For smaller, independent pharmacies, however, this ‘margin’ tends to make up most of their income and if these margins decrease, their very existence may be threatened. Furthermore, the challenge for UK pharmacies has been that an increase or introduction of fees for pharmaceutical services has been linked to a reduction in dispensing fees.

In Sweden, the pharmacy margin is similarly regressive; therefore, pharmacies receive higher compensation for higher priced products, but only up to a ceiling [28]. In Spain, although there is also a statutory regressive margin scheme for all outpatient medicines, community pharmacies have to make “claw back” payments to the national governmental health service based on their annual sales [11]. Case studies from Europe show that even in countries with comparatively high investments in health, offering sufficient remuneration not only for medicines but also for any related and additional clinical service is still a hard balance to strike [29].

### Challenge 2: incentivizing equitable access to medicines

Strategies to incentivize the use of retail pharmacies in less wealthy or less populated areas are one way to allow sufficient equitable access. Yet, Brazil’s FPP provides an important lesson: the inclusion of more retail pharmacies does not necessarily result in the desired level of equity in geographical access. Although the FPP first used public pharmacies to dispense a list of subsidized medicines that were purchased by the federal government, the program was broadened by contracting accredited, retail pharmacies to allow for greater expansion and geographical coverage. Including accredited private pharmacy outlets in the FPP led to a considerable increase in the number of pharmacies, but the geographical distribution was still skewed towards the wealthier South and Southeast areas of the country, and thus equity concerns remained [30].

Sweden makes funds available to support rural pharmacies, and Scotland, Northern Ireland and Wales guarantee minimum monthly pharmacy incomes [12]. In Spain, access to new licenses for retail pharmacies entails public tendering. However, once the pharmacist has won the license for a specific location, the license becomes a commodity and this sets up a market for pharmacies/licenses, though only another pharmacist can buy a retail pharmacy [13].

The evidence suggests that arrangements for retail pharmacy funding that rely solely on distance from one pharmacy to another as the means of determining funding allocation are ill-advised, because this could penalize community pharmacies in the most deprived communities and potentially have a negative effect on other healthcare providers, such as general practitioners, accidents and emergency services [31]. In Spain, equity and access to pharmacies seem to be better achieved by establishing demographic or needs-based criteria to open new pharmacies including “farmacia-botiquin” (pharmacy outlets) in rural areas, i.e., where pharmaceutical services are provided by one of the pharmacies in the nearer towns or villages [12, 32, 33]. Amongst those nearby pharmacies, one of them is designated as the only one eligible to cater to that portion of the population that is geographically isolated [32, 33].

### Challenge 3: ensuring quality of care and delivery of services

Scaling up the delivery of services and products by contracting retail pharmacies also requires enhanced supervision and inspection capabilities. There are opportunities to use digital technology to lower the costs of in-person inspections and supervisions. However, there is much room for development, and current systems in many LMICs are underfunded in this regard. LMICs considering contracting retail pharmacies should have supervision and inspection efforts specified in the pharmacy contracts themselves. These efforts should include the presence of technically responsible staff on-site and infrastructure for monitoring, such as computerized systems. However, this is often not enforced effectively. For instance, Brazil’s inclusion of a broad and consistent information system was comparable with those found in HICs and enabled the regulation of maximum dispensing amounts. However, the system has some transparency problems, notably information only becoming available recently, and important measures, such as the proportion of prescriptions originating in the public system, are not being reported. [24, 34]. Argentina does not have a framework for collaboration between regulatory entities and pharmaceutical organizations—seriously hampering the ability to enforce private pharmacy quality standards [35]. Ghana and Namibia also face challenges, mainly due to the scarcity of financial and human resource (HR) capacity and of sophisticated information systems [20, 36-38].

The accreditation process for pharmacies should also form part of a contract or agreement with the public payer [39]. The accreditation process often focuses on checking for inputs, such as facility size, supplies, and equipment. The process often oversees the assessment of the skills required to deliver services beyond initial professional licensure [40, 41]. For example, accreditation requirements were created when the Brazilian FPP was extended to the private pharmacy sector [24].These included a Health Surveillance Functioning Authorization, compliance with commercial regulations, the presence of a certified technically responsible pharmacist, fiscal capability, and infrastructure for a computerized system to issue invoices and receipts registration with the MoH. Despite its achievements in improving access, the FPP has been audited a number of times due to issues that compromise service quality, such as weak internal and administrative controls and lack of monitoring [30].

### Challenge 4: task-sharing from primary care providers to pharmacies

Dispensing and administering EMs may require special training. For instance, injectable contraceptives that must be administered intramuscularly are often provided in health facilities, whereas many client-initiated forms of contraception such as emergency contraception, combination oral contraceptives, progestin-only contraceptives, diaphragms or cycle beads tend to be provided at retail pharmacies [42, 43]. Task-sharing policies may allow trained community health extension workers and pharmacy staff to administer injectable products. This task sharing with lower-level trained providers presents an opportunity to expand access points to pharmacies [10]. However, it should be noted that such task sharing programs are not trivial to implement, because they would require significant regulatory reforms, extensive training programs and the establishment of supervision and inspection systems [44].

In HICs, there seems to be growing recognition that moving towards patient-centered pharmacy services may improve access to services [46-49]. Perhaps the best example of this is Sweden’s pilot program in developing clinical counseling by community pharmacies [48]. Those in favor point to increased competition between retail pharmacies and primary care points, leading, in principle, to better service to the public and more financial sustainability by attracting and keeping patients [28]. However, if pharmacy customers only know about the pharmacy's medicine-dispensing services, and no other healthcare services, it may well be difficult to build a pharmacy brand on this basis. Furthermore, determining the appropriate level and providing adequate remuneration for such services has been challenging [49, 50].

### Challenge 5: human resource and capacity constraints

Studies have shown that many pharmacy staff have poor knowledge of medicines and associated practices, especially for FP, including contraceptives [52-54]. In Ghana, fewer than 50% of pharmacy staff attend courses on appropriate medicine use, management, and Standard Treatment Guidelines (STGs) [38]. A 2016 systematic review of the performance of pharmacies across Asia’s LMICs found discrepancies between knowledge and actual practice, suggesting that these gaps cause the mismanagement of patients [54]. Similar issues were observed in Namibia, where development partners have been supporting the government with long-range planning of pharmaceutical HR and building capacity of training institutions for pre-service and in-service pharmaceutical management training [36].

One way to support a more efficient use of existing HR capacity is to leverage digital tools. One example is Maisha Meds, which, among other aspects of expanding access to medicines, offers digital tools that help providers better manage their pharmacies, pharmacy staff support in the supply chain, and pharmacy management topics [55]. For instance, pharmacies can order medicines through the Maisha Meds platform, which leverages sales data to design “smart” order packages based on prior sales volumes. In addition, Maisha Meds improves medicine access by leveraging the pharmacy point-of-sale (POS) system as a digital reimbursement platform to help global health funders and pharmaceutical companies subsidize products for high-risk or low-income patients. This latter capacity is especially noteworthy for the provision of medicines, such as FP, which are often understocked by retail pharmacies [55].

## Discussion

The findings from the case studies describe the key considerations, opportunities, and challenges for public payers related to contracting retail pharmacies. We provide examples of strategies and policies to best address these challenges. While public payers must ensure that contracted retail pharmacies have the capability to provide safe, quality-assured medicine and pharmaceutical services, they must have the capacity to develop, implement, and enforce these contracts. In some cases, this will require changes to the legal framework that underpins the operations of both public payers and retail pharmacies [56, 57]. Strategic policies may also require professional associations to change the way they oversee pharmacies and individual professional standards [57].

With respect to the specific challenges mentioned herein, any public–private sector contractual relationship must be able to continually balance how responsibility for service delivery can be shared between the public payer and the retail pharmacies, so that the retail pharmacies as businesses remain a profitable or attractive option. Yet the public payer must also remain financially viable and accountable for safeguarding a comprehensive UHC service delivery system that ensures equitable access to affordable EMs.

In addition to case studies, other literature describes strategies to attract pharmacies and enable them to purchase medicines at a lower price than those purchased from manufacturers’ distributors [59-61]. In some countries, private retail pharmacies have successfully participated in the national public procurement and supply chain, enabling pharmacies to benefit from lower purchasing prices—resulting in a higher pharmacy profit margin (reimbursement price minus pharmacy purchasing price) [61]. Other methods to enable lower pharmacy operating costs include improving inventory management, having wholesalers largely finance medicine stocks, and/or enabling private sector group purchasing organizations (GPOs) [60, 61, 63, 64].

The case studies further demonstrate that public payers planning to contract retail pharmacies will need to take the local supply chain structure into consideration. To do this effectively, a thorough market study of a country’s supply chain maturity is essential to help determine the public sector’s capabilities and performance weaknesses. For instance, Ghana’s national health supply chain system was assessed in 2019 in a joint effort by the Ministry of Health (MOH), Ghana Health Service, and USAID. Although the focus was on the public sector, the assessment provided details on the current state of Ghana’s health supply chain in terms of its capability, functionality and performance [64]. Although not systematically used across the globe, such situational analyses recognize the importance of the country context in determining which new contracting models are to be implemented and how they would interact with the entire supply chain [65].

It is equally important to keep in mind the specific stakeholders who are likely to benefit from extending medicine provisions via retail pharmacies. Public payers could add questions to routine household surveys such as the National Demographic and Health Survey (DHS) to identify unmet household needs in terms of access to medicines. The results can be informative in allowing the tailoring of private retail pharmacy services to such unmet medical needs.

These findings also have implications for contracting retail pharmacies during pandemics. For instance, one implication of this rethinking may be a shift away from in-person consultation services to telehealth services that providers plan to maintain in the coming years [66]. In addition, contracted pharmacies may need to offer delivery services or come to an agreement with third parties to have their medicines and other delivered/couriered products delivered to their customers’ homes or close to their homes [67].

Ensuring product quality requires an appropriate legal foundation to regulate both pharmaceutical products (to prevent falsification and substandard quality) and pharmacy practices (to ensure a minimum standard of pharmacy service). Furthermore, effective tools are needed to enact oversight, monitoring, and reporting strategies and allow the public payer to verify compliance with the contractual arrangements and reimbursement for services. The lack of such systems may lead to discrepancies in health system management performance and, consequently, to inequalities in population health outcomes.

The need to potentially task share from primary care providers to pharmacies will also require significant legal reforms, extensive training programs, and the establishment of supervision and inspection systems if they decide, for example, to provide permission and allow retail pharmacies to administer drugs, such as long-acting contraceptives. The implementation of such programmes is not trivial [44].

Public payers contracting retail pharmacies will need to rigorously evaluate existing relevant contracts, as it is critical to learn from their achievements to make the necessary adjustments to improve their performance. Effective oversight, monitoring, and reporting strategies are required to manage and evaluate the contracts. While HICs have contracted private pharmacies for decades and IT systems have had decades to mature, many governments in LMICs have not yet contracted with third parties for the provision of services at a large scale and do not necessarily have IT capacity to enable efficient dispensing verification, invoicing, and payment, all of which are critical to efficiently contracting these services. The examples of Brazil and Ghana show the need for constant policy readjustments to optimize their performance. Tapping into these IT platforms brings important benefits but also requires governments to rapidly scale up their applications. Many mobile phone applications already allow pharmacies to maintain constant communication to exchange information. It is important to note that although a step in the right direction, not all functionality is able to get onto a mobile application, and not all LMICs have access to good network coverage. Moreover, there is a need for more HR in health IT and a necessary legal framework for such systems.

The present work is not without its limitations. This is primarily a desk review with few in-person contacts and no qualitative work using focus groups, which would have provided some nuanced interpretations. We performed a scoping review and had no access to unpublished administrative reports. However, all case studies were reviewed by country experts for factual accuracy and the completed report was reviewed by three external reviewers. For this reason, we believe the views expressed herein should be able to serve as a framework within which to initiate a conversation among the relevant public and private stakeholders. We also believe that there is a need to support the implementation—on a pilot basis—of these contracting models in interested LMICs that are currently rolling out UHC programs to provide first-hand evidence and learnings for the future.

## Conclusions

The case studies and the complementary literature show that public sector contracting of retail pharmacies—when facilitated by IT—could offer significant opportunities to increase access to medicines. It is essential that donors and governments alike invest in evaluating these new business models, e.g., financing of medicine stock by wholesalers and decoupling of dispensing from service provisions, and in bringing them to scale if they successfully want to meet their objectives.

Not everything can be “resolved by contract.” The relationship between public payers and retail pharmacies must be specific to the context and evolve over time. Brazil and Ghana had to make a series of adjustments over more than a decade to carefully calibrate strategies that were appropriate for both the large health system and the market sector context. This balancing is, in our view, the key to understanding how public payers can effectively enter sustainable contractual partnerships with retail pharmacies.

## Data Availability

The data sets used and/or analyzed during the current study are available from the corresponding author upon reasonable request.

## Appendix 1: Literature search and review strategy

Figure 1 illustrates the methodology employed for this work.

### Databases and search terms

The literature search was conducted on PubMed, Google Scholar, and CINAHL and consisted of several topics related to retail pharmacies and public payers using a combination of medical subject headings (MeSH) and free text searches. For the Google Scholar search, we primarily used free-text searches based on the key concepts of interest. The search included the following limits: publication date from 2010 to present, English, Portuguese, and Spanish Language.

The following search terms were applied:

1. Google and Google Scholar Search: first 50 cites/then ‘cited by”, then ‘related references”
  - Search terms = public private partnership/family planning public sector/payer/private pharmacy AND (Argentina, Brazil, Ghana, Namibia, Spain Sweden, United Kingdom)
2. PubMed
  - ((cooperation, public private sector [MeSH Terms]) AND (medicine [MeSH Terms])) AND (family planning [MeSH Terms] AND (Argentina, Brazil, Ghana, Namibia, Spain Sweden, United Kingdom)
  - ((cooperation, public private sector) [Title/Abstract] AND (medicine)) [Title/Abstract] AND (family planning) [Title/Abstract] AND (Argentina, Brazil, Ghana, Namibia, Spain Sweden, United Kingdom)
3. CINAHL
  - AB (public AND private AND (cooperation OR partnership)) AND AB pharmaceutical AND (Argentina, Brazil, Ghana, Namibia, Spain Sweden, United Kingdom) AB= Abstract

In some instances, country experts and the MTaPS/USAID team members provided additional references that were not included in the original literature search. We carefully reviewed each of these references and added or updated information where relevant to enrich this paper.

### Data extraction and analysis

Once the relevant literature was identified, all authors reviewed the data to develop seven country-specific case studies. Each case study was divided into two parts. The first part was a brief description of the four aspects of the countries’ retail pharmacies, followed by the questions:

- How are retail pharmacies chosen by the public payer?
- What services are retail pharmacies contracted to offer?
- How are retail pharmacies paid for their services? Are there any incentives to offer high-quality services?
- How are retail pharmacies accredited, regulated, monitored, and audited?

The second part of the case study was a synthesis of the documents that assessed, monitored, and evaluated the impact of policies and strategies on contracting private pharmacies’ performance of the pharmaceutical series related to geographical accessibility, quality of pharmaceutical services, affordability, and access to family planning.

To verify factual accuracy, each country case study was reviewed by country-specific experts, all of whom provided feedback to be incorporated into the final version of each case study.

Once the case studies were reviewed by country experts and finalized, we extracted the advantages and disadvantages of contracting retail pharmacies to dispense medicines to public sector clients. For this purpose, we focused on the advantages and disadvantages of such contracting arrangements for the public payer, clients, and the private sector.

This was followed by a thorough analysis of the key considerations that must be made when deciding whether and how to contract retail pharmacies. To guide this analysis, we developed a framework which merged the general considerations needed by PPPs when contracting the private sector (2012 Work conducted by Barbara O’Hanlon as Principal Investigator and funded by USAID/SHOPS), with the specific considerations identified by the World Health Organization in its 2017 “Partnering with the private sector to strengthen provision of contraception” document [10]. Six key dimensions were identified: (1) governance, including regulation; (2) contracting and reimbursement; (3) affordability; (4) quality of care; (5) accessibility; (and 6) patient-centered pharmaceutical care). Table 2 describes our reasoning behind why each dimension is relevant in the context of contracting the private sector. We assessed each dimension for each of the seven country cases selected. The question that we asked was ‘how does the public sector payer in our case studies incorporate provisions and use strategies to achieve its goals with regard to each dimension?

**Table 2:** Key dimensions of contracting the private sector to deliver EMs

Dimensions	Explanation of why each dimension is relevant in the context of contracting the private sector
Governance including regulation	To ensure that governance and associated regulations support private sector provision of EMs/FP and that the private sector complies with public sector health standards and private sector business standards To facilitate effective governance, regulations, and reimbursement via transfer of electronic data between contractor and provider
Contracting and reimbursement	To guarantee timely and efficient service provision by the private sector and the transfer of funds by the public sector payer partner(s)
Affordability	To protect against financial hardship by the end user and the sustainability of the public payer
Quality of care	To maximize health benefits and use resource efficiently through support for patient-centered pharmaceutical service standards, medicine use reviews, and accreditation schemes for private sector pharmaceutical outlets
Accessibility	To promote equity in accessing retail pharmacies
Patient centered pharmaceutical care	To increase availability of services and greater provider choice and to improve patient satisfaction, adherence, and outcomes through better patient–provider communication techniques to reduce barriers to information access for pharmacies and patients To support private sector pharmacies, professional associations, consumer/patient advocacy groups, and civil societies to develop/adapt standards, guidelines, and tools to institutionalize pharmaceutical care as part of essential packages of health services to optimize patient use of medicines

Finally, we compared and contrasted the findings of the case studies and drew conclusions and recommendations for the public payer contracting of retail pharmacies. More specifically, we described a series of promising policies and strategies that the public payers in the cases studied have used and discussed how they have addressed the related challenges with complimentary policies and strategies. Contextual factors were considered, such as the wholesaler and distributor market, business regulations, and incentive structures, and the most recent developments stemming from the emergence of COVID-19 and other new and existing infectious diseases on the ability of public payers to contract retail pharmacies.

## Abbreviations

FP: Family planning
EM: Essential medicines
LMIC: Low- and middle-income country
UHC: Universal Health Coverage
USAID: United States Agency for International Development
HIC: High income countries
FPP: Farmácia Popular Program (Brazil)
MoH: Ministry of Health
HR: Human Resources
GPOs: Group Purchasing Organizations
DHS: Demographic Health Survey
IT: Information Technology
STG: Standard Treatment Guidelines
AFP: Aqui hà Farmacia Popular (Brasil)
NHIS: National Health Insurance Scheme (Ghana)
NHS: National Health Service (United Kingdom)
UK: United Kingdom
POS: Point of Sale
PSAO: Pharmacy Service Administration Organizations
